# Knowledge, attitude, and associated factors towards mental illness among residents of Dessie town, northeast, Ethiopia, a cross-sectional study

**DOI:** 10.1186/s12888-021-03609-0

**Published:** 2021-12-09

**Authors:** Mengesha Birkie, Tamrat Anbesaw

**Affiliations:** Department of Psychiatry, College of Medicine and Health Science, Wollo University, Dessie, Ethiopia

**Keywords:** Knowledge, Attitude, Mental illness, Factors, Dessie, Ethiopia

## Abstract

**Background:**

Knowledge and attitude towards mental illness are poor and neglected as compared to medical illness. However, there is an increasing burden of mental illness in the community. As per the knowledge of the investigators, studies are scarce and not done in the study area about knowledge and attitudes of mental illness. Therefore, this study aimed to assess the knowledge, attitude, and associated factors towards mental illness among, Dessie town residents.

**Methods:**

A community-based cross-sectional study was conducted in Dessie town, Northeast, Ethiopia from October 27 to November 29/2020. A multi-stage sampling technique was employed. The data were collected from 477 study participants using a standard structured questionnaire, which were Mental Health Knowledge Schedule (MAKS) and Community Attitude to Mental Illness Inventory (CAMI) respectively. Data were entered using Epi-data version 3.1 and, then exported to SPSS version 26 for analyses. Bivariable and Multivariable logistic regression analyses was employed.

**Results:**

From 477 study participants the prevalence of poor knowledge and unfavorable attitude towards mental illness were 55.3% (95% CI: 50.9, 60.0) and 45.1% (95% CI: 40.7,49.5) respectively. Being female [AOR = 1.62 (95% CI:1.06,2.47)], could not read and write [AOR = 6.28 (95% CI: 2.56, 15.39)], lack of information about mental illness [AOR = 5.82 (95% CI: 3.78,8.94)] and unfavorable attitude [AOR =1.73 (95% CI: 1.12,2.66)] were variables found statistical significant with poor knowledge. Whereas, income < 2166 [AOR = 1.64, (95% CI: 1.12, 2.41)], poor social support [AOR = 2.04, (95% CI: 1.13, 3.68)], moderate social support [AOR = 2.44, (95% CI: 1.45, 3.97)] and poor knowledge [AOR = 1.66, (95% CI: 1.13,2.43)] were variables significantly associated with unfavorable attitude toward mental illness.

**Conclusion:**

In this study about half of the community have poor knowledge, and less than half of the participants have an unfavorable attitude to wards mental illness. There are many factors associated with poor knowledge and unfavorable attitudes. This having poor knowledge and unfavorable attitude may cause certain problems like a decrease in health care of a person with mental illness. Therefore, we recommend practice-based awareness in the community regarding mental health problems needs to be addressed.

## Background

Mental illness is a term used to describe a broad range of mental and emotional disturbances affecting individuals thinking, feeling, decision making, mood, daily functioning as well as ability to relate to others [1, 2]. According to Gill, causes of mental illness are not synonymous, but vary widely, from inherited chemical imbalances accountable for the development of such illnesses as depression and bipolar disorder [3]. Globally, 450 million people are predicted to be suffering from psychiatric disorders. However, in our day-to-day activity mental, physical, and social health are the necessary components in every individual [4]. In addition, according to a WHO report, globally more than 25% of people are experienced mental illness at some point in their lifetime. The Nationwide Institution of Mental Health (NIMH) in the United States also estimates that 1 in 5 people (20%) would be affected by this condition at some point in their lives [5].

Currently, mental illness is a public health problem in developed as well as developing countries [6]. However, studies suggest that some mental disorders are even common in low income than in developed, it is estimated by the year 2000 there would be 24.4 million schizophrenia in less developed countries [6, 7]. The prevalence of poor knowledge is significantly very high in deferent countries, in Qatar 72.5% [8], in Saudi public 87.5% [9], in Tanzania 85.9% [10], in Kinondoni 61% [3], in Nigeria 51.2% [11], in Moroccan 76% [12], and in Ethiopia 59.2% [13]. Whereas unfavorable attitude, in Tanzania 58.9% [10], in Kinondoni 79.6% [3], Saudi public 54.5% [9] and Nigeria 10.0% [11].

In Ethiopia, there are widespread beliefs that severe mental illness is due to demon possession, or the evil eye has existed for many years, this influence the attitude towards mental illness [14]. In Ethiopia, where preventable infectious diseases are very widespread, has got great attention. But mental health problems, which are regarded as non-life-threatening problems, due to this, are not given attention. However, mental health problems account for 12.45% of the burden of diseases in Ethiopia and 12% of the Ethiopian people are suffering from some form of mental health problems of which, 2% are severe cases [15, 16].

Previous studies reported that there are several identified factors associated with poor knowledge and unfavorable attitude, have low educational attainment, unemployment, age, sex, poor levels of social support, low income, absence of relative with a mental disorder, unemployed history of mental illness who do not have better information access to mental illness [3, 8, 9, 11, 13, 17–25]. About 14% of the global burden of disease have been attributed to mental and related disease, mostly chronically disabling illness, depression and other common mental disorders like psychosis [26]. Of all the health problems, mental illnesses are poorly understood by the universal in the community. Such poor knowledge and negative attitude towards mental illness threaten the success of patient care, rehabilitation, the healing process, unable to use effective treatment, lead to stigmatization, inhibit help-seeking behavior and provide proper holistic care [4, 5]. Furthermore, negative attitudes and discrimination deprive victims of human dignity and prevent social participation. These negative experiences decrease self-esteem and instill feelings of shame and guilt [26].

Despite mental illness is a significant problem globally, especially in Dessie town,Ethiopia [27],and it is on the rise associated with deep-rooted little knowledge and negative attitude toward mental. Even, if there are several studies done on knowledge and attitude towards mental illness in Ethiopia [13, 28–30]. To the best of our knowledge, no study assessed the community knowledge and attitude towards mental illness in Dessie town residents, Ethiopia. Therefore, this study finding will have added greatly to the current knowledge by giving appropriate evidence that will be significantly important for health planners, decision-makers, future researchers, and politicians.

## Methods and materials

### Study area, design, and period

A Community-based cross-sectional study was conducted from October 27 to November 29/2020 in Dessie town, South Wollo zone, North East, Ethiopia. The town is geographically located 401 km from Addis Ababa to the northeast part of Ethiopia. It has five sub-town with a total population of 350,000. Among those populations 186,571 males and 163,429 females according to 2016 to 2017 South Wollo Zone statistics office data.

### Source of population

All residents of Dessie town, North East, Ethiopia.

### Study population

Households live in the Menafesha sub-town who are in the selected kebele and available during the study period.

### Inclusion and exclusion criteria

#### Inclusion criteria

All households in selected kebeles,who was > 18 years old, and participants living > 6 months in Dessie Town during the time of the study were included.

#### Exclusion criteria

Participants who were severely ill and unable to communicate during data collection time were excluded from the study.

### Sample size determination and sampling technique

#### Sample size determination

To calculate the maximum estimated sample size, single population proportion formula was used at 95% CI and 5% marginal error and by taking the *P*-value of 59.2% from the previous study in Mekelle town residents [13]. Then the sample size was calculated as follows.

$$n=\frac{{\left(Z\ a/2\right)}^2\ P\left(1-P\right)}{d^2}$$

Where: n = sample size = prevalence of previous study (59.2%), d = degree of precision (assumed to be 5%), (z α/2)2 = 1.96,confidence levels at (95%).

$$n=\frac{(1.96)^2\times \left(0.592\ \left(1-0.592\right)\right.}{(0.05)^2}=371$$

Since we have employed a multi-stage sampling technique, by considering the designing effect, the calculated sample size was multiplied by 1.5 to correct the sampling error. After all, by adding a 5% non-response rate, the final sample size was 584.

#### Sampling technique and procedure

**Fig. 1:**
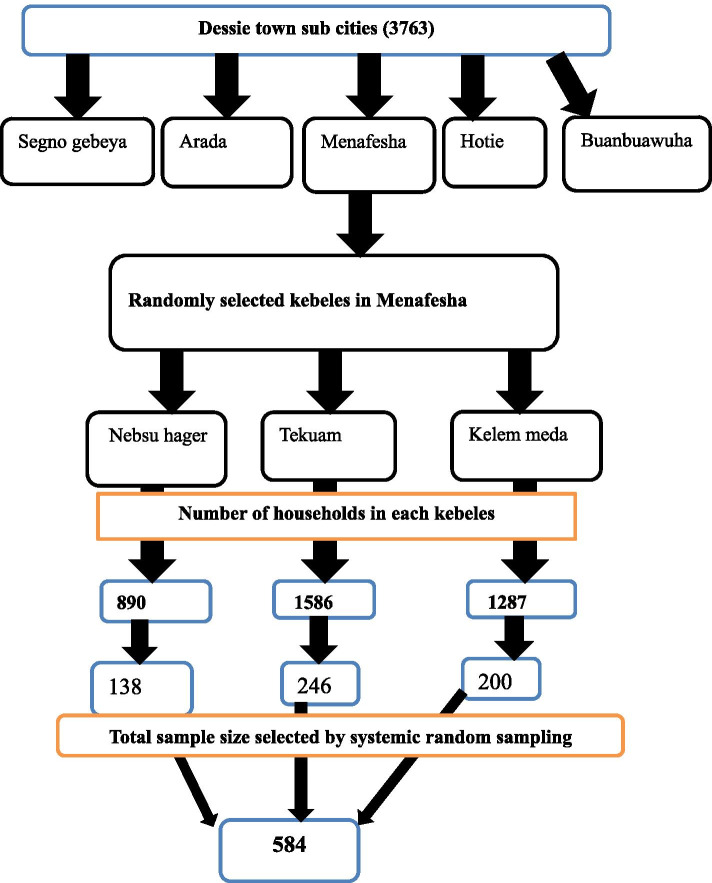
schematic representation of sampling technique showing the number of selected samples from each sub-city of Dessie town, 2020 G.C

A multi-stage probability sampling technique was employed. There are five sub-cities in Dessie town, and one sub-town was selected from the total, using a simple random sampling technique, then within this chosen sub-town there are three kebeles, and the numbers of participants were selected from each kebele using the proportional allocation of the sample size. Then, a systematic random sampling technique was employed to select study units. The first study unit was selected randomly between 1st and K^th^ then study subjects were selected in every K^th^ household. When more than one study subject was found in one household, a lottery method was used to select a participant. If the selected house is closed during data collection, the next household participants were interviewed. i.e. K_j_ = N_j_/n_j_ Where K_j_ = sampling interval, N_j_ = total number of households, n_j_ = total number of sample size. There are three kebeles in the Menafesha sub-town and having a total of 3763 households, K or interval was determined by N/n (K = 3763/584 = 7). We were interviewed one individual from every 7 households (Fig. 1).

### Data collection method and technique

Data were collected using an interviewer-administered questionnaire. It includes socio-demographic variables, clinical variables, psychosocial factors, and outcome variables are knowledge and attitude towards mental illness. The Mental Health Knowledge Schedule MAKS (6 items) is a standardized instrument adapted and used for this study to assessing knowledge of the mental illness. The five-point scale was used to show total disagreement (scale = 1) and strong agreement (scale = 5). Better mental health literacy was shown by higher scores [31]. To determine the knowledge toward mental illness, similar to another study it became dichotomized [18]. For the cut point, we categorized the knowledge of those respondents with a mean value of 20.23 (poor knowledge < 20.23). In this study, the Cronbach α of the MAKS items was showed 0.71.

Attitudes towards mental illness are measured by the Community Attitude to Mental Illness Inventory (CAMI). It has 4 items with four sub-scales which are authoritarianism, benevolence, social restrictiveness, and community mental health ideology. All items are rated according to a 5 Likert scales (1 = strongly disagree, 2 = disagree, 3 = neutral, 4 = agree, 5 = strongly agree). It has been used in various countries of Africa including Ethiopia [29]. We categorized respondents as with favorable and unfavorable attitudes based on the mean score which was 117 (Good < 117). Social support was assessed by the Oslo social support 3 item questions with three categories; a score of 3–8 is poor social support, moderate social support 9–11, and strong social support 12–14 [32].

### Data quality contro**l**

The questionnaire was prepared first in English and translated into the Amharic language then back-translated to English to check the consistency. Furthermore, we use standard tools to determine the outcome variable by giving 2 days of training for data collectors and supervisors. The pre-test was conducted with 5% (*n* = 28) of the participants in the Buanbuha sub-Town to identify potential problems in data collection tools and modification of the questionnaire. Regular supervision and support were given for data collectors by the supervisor and principal investigator. Data were checked for completeness and consistency by supervisors and principal investigators daily during data collection time for its completeness.

### Data processing and analysis

The data was entered into the Epi-Data version 3.1, and then data was exported to SPSS 25.0 version for cleaning and analysis. Descriptive statistics: means, frequency, percentages, and standard deviations were calculated and presented in tables and graphs. Independent predictors were determined using logistic regression modeling. All variables *p*-value < 0.25 in the bivariable analysis were entered into the multivariable logistic regression model. The model of fitness was checked by Hosmer and Lemeshow goodness. A *P*-value < 0.05 was considered statistically significant and the strength of the association was measured by an odds ratio of 95% C.I.

## Results

### Socio-demographic distribution of the participants

This study intended to be included 584 households, despite 477 participated in the study which makes the response rate of 81.67%. The mean age (±SD) of the respondents was 36.26 (±11.7) years, with an age range of 18–70 years. From the total participants, the majority 257(53.9%) were male. The largest proportion of the participants were married 284 (59.5%), Amhara 470 (98.5%), and Muslim 358 (75.1%) in their respective domains. Besides, nearly one third of the participants were merchants 160 (33.5%). . More than half of 268 (56.2%) participants reported that their average monthly income was >=2166 Ethiopian birr (Table 1).

**Table 1:** Distribution of socio-demographic factors of Dessie town residents, Ethiopia, 2020 (***N*** = 477)

Variable	Categories	Frequency	Percent (%)
Age in years	18–24	81	17.0
	25–34	173	36.3
	35–44	134	28.1
	> 44	89	18.6
Sex	Male	257	53.9
	Female	220	46.1
Marital status	Married	284	59.5
	Single	139	29.1
	Widowed	33	6.9
	Divorced	21	4.4
Ethinicity	Amhara	470	98.5
	Other^a^	7	1.5
Religion	Muslim	358	75.1
	Orthodox	97	20.3
	Protestant	22	4.6
Level of education	Unable to read and write	62	13.0
	Primary school	73	15.3
	Secondary school(9–12 grade)	112	23.5
	College diploma	111	23.3
	University degree and above	119	24.9
Occupation	Government employee	122	25.6
	Housewife	78	16.4
	Merchant	160	33.5
	Student	71	14.9
	Others^b^	46	9.6
Monthly income	< 2166 ETB	209	43.8
	> = 2166	268	56.2

Others^a^:-Oromo & Tigre, ^b^:- Retired, NGOs & Farmer, ETB = Ethiopian birr

### Source of information about mental health, clinical and psychosocial factors

Among the respondents, 252 (52.8%) had information about mental illness within the last year. Regarding the source of information, 126 (50%) of them were received from mass media. Of the study participants, 58 (12.2%) had a mental illness. All of the respondents know a mentally ill person through their life. Regarding their relationship knowing mentally ill person 106 (22.2%) were relatives, 142(29.8%) were neighbors, 58 (12.2%) were friends and 171(35.8%) of them were know with no relation. Out of the total participants, most of them, 305 (63.9%) respondents were never involved in caring for a person with a mental illness. Nearly one-third of participants 143 (30.0%) were threatened or hurt by a mentally ill person. Besides, 217 (45.5%) of residents witnessed others being hurt. The majority of the study participants 265 (55.6%) had received middle social support (Table 2).

**Table 2:** Description of information source about mental health, clinical and psychosocial related factors among Dessie town residents 2020 (*N* = 477)

Variable	Categories	Frequency	Percent(%)
Last 1 year, get information	Yes	252	52.8
	No	225	47.2
Source of information	Mass media	126	50.0
	Health institution	96	38.0
	Religious institution	30	12.0
Mental illness history	Yes	58	12.2
	No	419	87.8
Know somebody who has/had mental illness	Yes	477	100.0
	No	0	0.0
Relationship to the person with mental illness	Relative	106	22.2
	Neighbor	142	29.8
	Friends	58	12.2
	Person without relation	171	35.8
Caring for a person with mental illness	Yes	172	36.1
	No	305	63.9
Threatened or get hurt by mental illness person	Yes	143	30.0
	No	334	70.0
Have you ever seen a mental health apprehension or abuse victim	Yes	217	45.5
	No	260	54.5
Social support	Poor social support	100	21.0
	Moderate social support	265	55.6
	Strong social support	112	23.5

### Knowledge and attitude toward mental illness among Dessie town residents

**Fig. 2:**
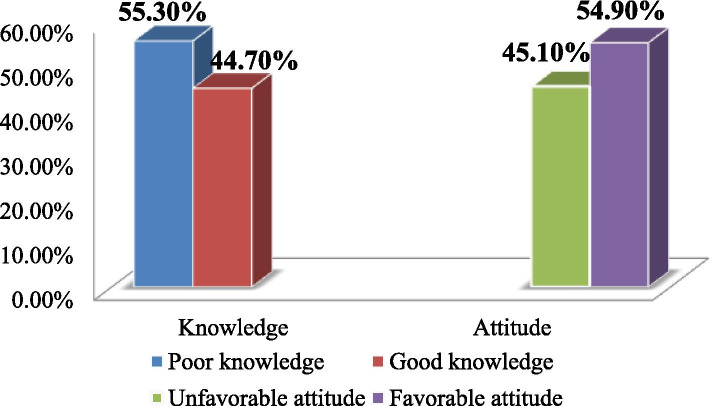
Magnitude of knowledge and attitude toward mental illness among Dessie town residents 2020 (N= 477)

Regarding the overall magnitude of knowledge of participants towards mental illness, about 264 [55.3% (95% CI,50.9, 60.0)] had poor knowledge and 215 [45.1% (95% CI,40.7, 49.5)] had unfavorable attitudes (Fig. 2).

### Help-seeking behavior of the study participants

In this study, from the total respondents 454 (95.2%) of them believe that they will get help from an intimate partner if they were having a mental or emotional problem, 403(84.5%) from the neighborhood, 422(97.7%) from parents, 467(97.9) from relatives, 456(95.6%) from health workers, 409(85.7%) from religious leaders and 195(40.9%) from traditional healers (Table 3).

**Table 3:** Frequency of help-seeking behavior among Dessie town residents 2020 (*N* = 477)

Variable	Categories	Frequency	Percent (%)
Intimate partner (husband,wife)	Likely	454	95.2
	Unlikely	23	4.8
Neighborhood/close friends	Likely	403	84.5
	Unlikely	74	15.5
Parents	Likely	467	97.9
	Unlikely	10	2.1
Another family member/relatives	Likely	450	94.3
	Unlikely	27	5.7
Health worker(e.g doctors, nurse, psychologist, counselor, social worker	Likely	456	95.6
	Unlikely	21	4.4
Religious leader	Likely	409	85.7
	Unlikely	68	14.3
Traditional healers	Likely	195	40.9
	Unlikely	282	59.1

### Factors associated with knowledge status towards mental illness

Factors associated with poor knowledge in the bivariable analysis such as, age, sex, marital status, educational status, information about mental health, suffered from any mental illness, social support, and unfavorable attitude showed a *p*-value of < 0.25 and became candidates for multivariable analysis.

In the multivariable logistic regression analysis, respondents who are female sex, cannot read and write, lack of information about mental health, and have an unfavorable attitude towards mental illness were variables statistically associated with poor knowledge about mental illness at *P*-value less than 0.05. Accordingly, those being female [AOR = 1.62 (95% CI: 1.06,2.47)],who could not read and write [AOR = 6.28 (95% CI: 2.56, 15.39)], lack of information about mental [AOR = 5.82 (95% CI: 3.78,8.94)] and unfavorable attitude [AOR =1.73 (95% CI: 1.12,2.66)] were variables found statistical significant with poor knowledge towards mental illness (Table 4).

**Table 4:** Bivariable and multivariable logistic regression analysis of associated factors for poor knowledge towards mental illness among Dessie residents, 2020

Variables	Category	Knowledge		COR(95%C.I)	AOR(95%C.I)	***P*** values
		Poor	Good			
Age	18–24	52(64.2%)	29(35.8%)	1.22(0.65,2.26)	2.08(0.99,4.37)	0.052
	25–34	84(48.6%)	89(51.4%)	0.64(0.38,1.07)	0.92(0.49,1.74)	0.811
	35–44	75(56.0%)	59(44.0%)	0.86(0.50,1.48)	0.98(0.51,1.89)	0.953
	> 44	53(59.6%)	36(40.4%)	1	1	
Sex	Female	138(62.7%)	82(37.3%)	1.75(1.21,2.52)	1.62(1.06,2.47)	**0.025**
	Male	126(49.0%)	131(51.0%)	1	1	
Marital Status	Married	161(56.7%)	123(43.3%)	1.29(0.86,1.93)	1.19(0.69,2.05)	0.533
	Widowed	20(60.6%)	13(39.4%)	1.55(0.70,3.28)	1.47(0.53,4.10)	0.457
	Divorced	13(61.9%)	8(38.1%)	1.60(0.62,4.11)	1.36(0.44,4.22)	0.587
	Single	70(50.4%)	69(49.6%)	1	1	
Educational status	Unable to read and write	54(87.1%)	8(12.9%)	8.69(3.81,19.87)	6.28(2.56,15.39)	**< 0. 000**
	Primary school	45(61.6%)	28(38.4%)	2.07(1.14,3.75)	1.73(0.87,3.42)	0.114
	Secondary school [9–12]	60(53.6%)	52(46.4%)	1.48(0.88,2.50)	1.04(0.57,1.89)	0.883
	College diploma	53(47.7%)	58(52.3%)	1.17(0.70,1.98)	0.89(0.49,1.62)	0.703
	University degree and above	52(43.7%)	67(56.3%)	1		
Any information about mental health	No	172(76.4%)	53(23.6%)	5.644(3.78,8.43)	5.82(3.78,8.94)	**< 0.001**
	Yes	92(36.5%)	160(63.5%)	1	1	
Suffered from any mental illness	No	237(56.6%)	182(43.4%)	0.55(0.27,1.13)	1.36(0.72,2.59)	0.345
	Yes	27(46.6%)	31(53.4%)	1	1	
Social support	Poor social support	63(63.0%)	37(37.0%)	2.11(1.22,3.66	1.63(0.84,3.14)	0.143
	Moderate social support	151(57.0%)	114(43.0%)	1.64(1.05,2.56)	1.493(0.88,2.52)	0.133
	Strong social support	50(44.6%)	62(55.4%)	1	1	
Attitude	Negative attitude	135(62.8%)	80(37.2%)	1.74(1.21,2.51)	1.73(1.12,2.66)	**0.013**
	Positive attitude	129(49.2%)	133(50.8%)	1		

1 = reference category, Chi square = 8, Hosmer Lemeshow goodness-of-fit 0.56

### Factors associated with unfavorable attitude towards mental illness among Dessie residents

In the bivariable analyses, marital status, income, suffered from any mental illness, social support, and knowledge showed a *p*-value of < 0.25 and became candidates for multivariable analysis. In multivariable binary logistic regression analyses, income, social support and poor knowledge were statistically associated with unfavorable attitude toward mental illness (Table 5).

**Table 5:** Bivariable and multivariablelogistic regression analysis of associated factors for unfavorable attitude towards mental illness among Dessie Town residents, 2020

Character		Attitude		COR(95%C.I)	AOR(95%C.I)	P-value
		Unfavorable	Favorable			
Marital status	Married	130(45.8%)	154(54.2%)	1.18(0.78,1.77)	1.45(0.93,2.26)	0.102
	Widowed	15(45.5%)	18(54.5%)	1.16(0.54,2.50)	1.18(0.53,2.63)	0.679
	Divorced	12(57.1%)	9(42.9%)	1.86(0.73,4.71)	1.87(0.71,4.95)	0.202
	Single	58(41.7%)	81(58.3%)	1		
Income	< 2166	110(52.6%)	99(47.4%)	1.72(1.19,2.48)	1.64(1.12,2.41)	**0.011**
	> = 2166	105(39.2%)	163(60.8%)	1	1	
Suffered from any mental illness	Yes	32(55.2%)	26(44.8%)	1.58(0.91,2.75)	1.73(0.97,3.07)	0.06
	No	183(43.7%)	236(56.3%)	1	1	
Social support	Poor social support	50(50.0%)	50(50.0%)	2.61(1.47,4.62)	2.04(1.13,3.68)	**0.018**
	Moderate social support	134(50.6%)	131(49.4%)	2.673(1.65,4.31)	2.44,1.45,3.97)	**< 0.0001**
	Strong social support	31(27.7%)	81(72.3%)	1	1	
Knowledge	Poor knowledge	135(51.1%)	129(48.9%)	1.74(1.20,2.51)	1.66(1.13,2.43)	**0.009**
	Good knowledge	80(37.6%)	133(62.4%)	1	1	

1 = reference category, Chi square = 8, Hosmer Lemeshow goodness-of-fit 0.32

## Discussion

Even if, the prevalence of mental illness growing rapidly in the world, especially in Dessie, Ethiopia, the knowledge, and attitude of people toward mental illness are not well addressed. Therefore, this study was intended to address this gap by assessing the knowledge and attitude towards mental illness in Dessie town residents in Ethiopia.

This study showed that about 55.3% of participants had poor knowledge and 45.1% had unfavorable attitudes towards mental illness. Regarding the level of poor knowledge, our result is in agreement with findings reported from Ethiopia 59.2% [13] and Nigeria 51.2% [11]. However, the prevalence in the current study is lower than the study conducted in Qatar 72.5% [8], Tanzania 85.9% [10], Kinondoni community 61% [3], Saudi public 87.5% [9], and Moroccan 76% [12]. This discrepancy might be due to variation in used inclusion criteria, in the academic qualification of the study participants, study period, study design, sampling size, tools, socioeconomic, sociocultural, and demographic characteristics.

On the other hand, in this study, 45.1% of respondents had reported an unfavorable attitude. The result is in line with a study conducted in Jimma, Ethiopia 46.9% [33]. But it is higher than the study done in Nigeria, the majority (90.0%) had positive attitudes toward mental illness [11]. The finding in the current study was lower than the result reported from Qatar which showed that the prevalence of unfavorable attitude was 53.5% [8], Tanzania 58.9% [10], Kinondoni 79.6% [3], and Saudi public 54.5% [9]. This variation might be due to numerous factors including differences in the characteristics of study participants, geographical location, socio-cultural variation, and the inclusion and exclusion criteria used.

The second objective of our study was to identify factors associated with poor knowledge and unfavorable attitude. A respondent with female in sex 1.62 times more likely to have poor knowledge as compared with males and this was supported by a study done in Ethiopia [13], Qatar [8], and India [19]. The possible explanation might be men are more eager to visit a psychiatrist for their emotional problems which might increase knowledge, while women preferred a traditional healer. Females are more afraid than males to talk about their illness. Furthermore, men obtained more information than women from the media; women favored healthcare staff more than men did [19].

Participants who could not read and write were 6.28 more likely to have poor knowledge as compared with a university degree and above and this was supported by a study conducted in Ethiopia [13, 23, 24], Malaysia [18], and Kinondoni [3]. It suggests that individuals with lower education had poor knowledge relating to mental illness. The possible explanation could be that participants with lower education have less access to health information, or they have a lesser ability understanding of such information as a result of their lower education. Furthermore, as recognized by World Health Organization that less education leads to lesser openness about mental illness [3].

Lack of information about mental health 5.82 more likely to have poor knowledge as compared to those who have information about mental illness. This study was a similar study conducted at Hawasa [28]. Reported that exposure to PWMI and having mental health information reduces stigma against mental illness leading to an overestimation of their information level and resulting from inadequate delivery of information by the public [15, 16].

Another factor associated with poor knowledge was unfavorable attitude, which was 1.73 times more likely to have a poor knowledge toward mental illness than a favorable attitude. Negative attitudes result in impeding requiring knowledge, which leads to discrimination, victims of human dignity, prevent social participation, decrease self-esteem, instill feelings of shame and guilt [26].

Participants with income < 2166 ETB have 1.64 more likely to have unfavorable attitudes as compared to favorable attitudes. This might be related to some negative emotions posed by survival pressure over low-income earners might result in an unfavorable attitude. Also, people with lower socioeconomic status have been hypothesized to get negative social, psychological, and economic skills that against the effect of hardship which would keep the negative attitude. This finding is similar to the study reported in Ethiopia [23] and the Saudi public [9].

Participants with poor knowledge had 1.66 times more likely to have unfavorable attitudes as compared to favorable attitudes. This is supported by a study done in conducted in Dodoma Municipality, Tanzania. lack of knowledge towards MI is significantly associated with attitude towards persons with mental illness [10]. In this case, possessing less knowledge of mental illness that individuals would have less social distancing, less tolerance/support to community care, and more social restrictiveness towards people with mental illness. In Ethiopia, most people beliefs that mental illness is due to demonic possession, bewitchment by the evil spirit and ancestors’ spirits, this influence the attitude towards mental illness and practice associated with mental health problems [14]. Another reason such as cultural variances might also play a significant role in this process [34].

The participant with poor social support were 2.04 times and moderate social support 2.44 times more likely to have unfavorable attitudes as compared to strong social support. This is the supported study conducted in Ethiopia. An individual who has experienced low social support has a poor attitude toward mental illness [13].

### Limitations

This study might lack the establishment of the temporal relationship between the dependent and independent variables, because of the cross-sectional nature of the study design. Furthermore, since it was collected by face-to-face interview method, it might prone to social desirability bias. Finally, it was conducted in only Dessie town the study population might not generalizable for other general populations.

## Conclusion

The finding of this study showed that, the knowledge and attitude of the community were poor, due to, different factors, such as being female, who could not read and write, lack of information about mental illness, and unfavorable attitude. Whereas, income < 2166, poor social support, moderate social support, and poor knowledge were factors to unfavorable attitude towards mental illness. This showed that a strong need, of community education program to increase awareness regarding mental illness, to improve access to psychiatric care for mentally ill persons, decrease stigma and unfavorable attitude towards mental illness.

## Data Availability

All data can be accessed from the corresponding author with the email address (mengeshasun@gmail.com).

## Abbreviations

AOR: Ajusted Odds Ratio
CAMI: Community Attitude Mental Illness
CI: Confidence Interval
CMHI: Community Mental Health Ideology
COR: Crude odds ratio
Epi-Data: Epidemiological Data
GGFRC: Gilgel Gibe Field Research Center
KA: Knowledge, Attitude
MASK: MentalHealth Knowledge Schedule
NGOs: Non-Governmental Organizations
PWMI: People with Mental Illness
SD: Standard Deviation
SPSS: Statistical Package for Social Sciences
USA: United States of American
WHO: World Health Organization
